# Stabilization and racetrack application of asymmetric Néel skyrmions in hybrid nanostructures

**DOI:** 10.1038/s41598-023-40236-z

**Published:** 2023-08-21

**Authors:** Mateusz Zelent, Mathieu Moalic, Michal Mruczkiewicz, Xiaoguang Li, Yan Zhou, Maciej Krawczyk

**Affiliations:** 1 Faculty of Physics, Institute of Spintronics and Quantum Information, Adam Mickiewicz University, Poznan, ul. Uniwersytetu Poznańskiego 2, 61-614 Poznan, Poland; 2 Institute of Electrical Engineering, Slovak Academy of Sciences, Dubravska cesta 9, Bratislava, 841-04 Slovakia; 3 Centre For Advanced Materials Application CEMEA, Slovak Academy of Sciences, Dubravska cesta 9, Bratislava, 845 11 Slovakia; 4 College of Engineering Physics, Shenzhen Technology University, Shenzhen, 518118 China; 5 School of Science and Engineering, The Chinese University of Hong Kong, Shenzhen, 518172 China

**Keywords:** Ferromagnetism, Magnetic properties and materials, Spintronics, Surfaces, interfaces and thin films

## Abstract

Magnetic skyrmions, topological quasiparticles, are small stable magnetic textures that possess intriguing properties and potential for data storage applications. Hybrid nanostructures comprised of skyrmions and soft magnetic material can offer additional advantages for developing skyrmion-based spintronic and magnonic devices. We show that a Néel-type skyrmion confined within a nanodot placed on top of a ferromagnetic in-plane magnetized stripe produces a unique and compelling platform for exploring the mutual coupling between magnetization textures. The skyrmion induces an imprint upon the stripe, which, in turn, asymmetrically squeezes the skyrmion in the dot, increasing their size and the range of skyrmion stability at small values of Dzyaloshinskii–Moriya interaction, as well as introducing skyrmion bi-stability. Finally, by exploiting the properties of the skyrmion in a hybrid system, we demonstrate unlimited skyrmion transport along a racetrack, free of the skyrmion Hall effect.

## Introduction

Magnetic skyrmions are stable nanometric-size spin textures with potential for memory, spintronic and magnonic applications due to the unique properties governed by their nontrivial topology^1–7^. The antisymmetric exchange energy term, known as Dzyaloshinskii–Moriya Interaction (DMI) helps to stabilise the skyrmion. It arises from spin-orbit coupling and inversion symmetry breaking. DMI can be observed in single-crystal materials such as B20-type crystals, leading to bulk DMI, or at the interface of thin ferromagnetic films with heavy metals, resulting in interfacial DMI. While DMI is favorable, it is not always essential^8^ for the formation and stabilization of Bloch and Néel type skyrmions. In addition to DMI, perpendicular magnetic anisotropy (PMA) also plays a crucial role in chiral magnetization textures stabilisation’s, especially in the case of thin films^9–11^. Both the static and dynamic properties of skyrmions are intensively studied in a wide range of materials and structures^3,4,12,13^. In particular, the stability of skyrmions at room temperature has been demonstrated in thin films and in confined geometries, like ferromagnetic stripes and nanodots^14–18^. Often, despite topological protection, the skyrmion configuration is not self-nucleating and is not the ground state^19–21^. Therefore, an important question arises whether the right selection of materials and nano-structuralization or environmental conditions can improve the skyrmion stability and provide effective functionality for applications.

Synthetic antiferromagnets offer an important advance in skyrmions’ principal application, i.e., as an information carrier in racetrack memory. In such bilayered tracks with opposite magnetization polarization, the pair of coupled skyrmions exerts opposite torques, allowing to mitigate skyrmion spin-Hall effect (SkHE) and providing a straight flow of the skyrmions^22^. From the application point of view, also elliptically deformed skyrmions show promising properties^23–25^. Here, the axis-symmetry breaking has been achieved by an in-plane bias magnetic field^26^ or by introducing a certain in-plane anisotropy^27^, for instance, by applying strain^27,28^. Recent studies show that also an anisotropic PMA and DMI can stabilize elliptical Néel-skyrmions in thin films^29,30^, that exhibit a considerable reduction of SkHE^31^.

**Figure 1:**
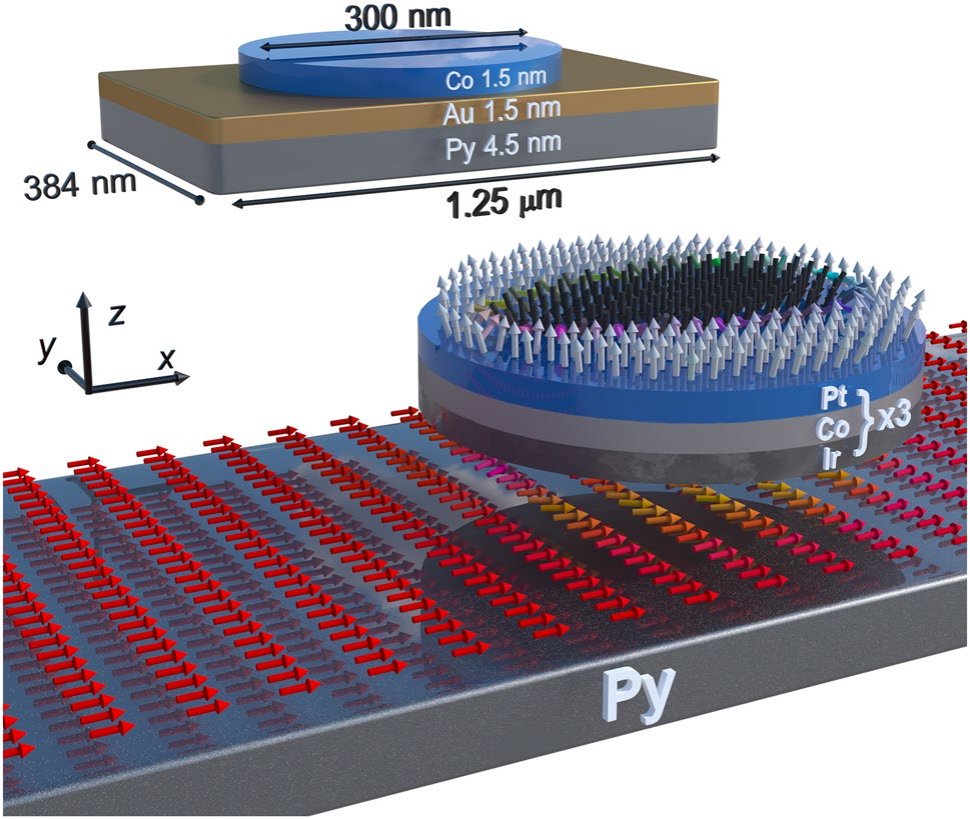
Visual representation of the system under consideration. Bellow, a 3D artistic visualisation shows the distribution of magnetisation in a Pt/Co/Ir multilayer and a Py stripe. Above, structure used in micromagnetic simulations: 1.5 nm thick Co nanodot separated by a 1.5 nm thick Au layer from the 4.5 nm thick Py stripe. In the dot, an egg-shaped Néel-type skyrmion is stabilized by the magnetostatic coupling to the skyrmion imprint upon the in-plane magnetized stripe. The arrows and their color (according to the HSL cone color scale) indicate the direction of magnetization. Note that the figure is not to scale.

We show that an egg-shaped Néel skyrmion with one axis of symmetry deformation can be stabilized by magnetostatic interaction in a hybrid structure composed of a multilayered nanodot hosting a skyrmion and the in-plane magnetized thin stripe made of soft ferromagnetic material, Fig. 1. The skyrmion state generates a nonuniform stray magnetic field, which affects the magnetization in the adjacent layer. The disturbed magnetization in the adjacent layer, an imprint, induces counter–stray magnetostatic field, which exerts a significant effect on the skyrmion static configuration, breaking its circular symmetry and enhancing effects similar to DMI. Importantly, this mutual interaction of the skyrmion and the stripe increases the skyrmion stability and opens a narrow range of the DMI parameter *D* for the bi-stabilization of the skyrmion. Moreover, the interaction also causes the skyrmion to grow in size, analogous to the effect of an external magnetic field^9,16,32,33^, thus allowing for skyrmion size control. Degree of the skyrmion’s asymmetric deformation can be influenced by several factors, including the strength of the magnetic anisotropy of the film, the polarity of the skyrmion or stripe, and the chirality of the skyrmion and also DMI strength. We demonstrate similar properties of skyrmions in a racetrack-type hybrid nanostructure, with proof-of-concept simulations showing the unconstrained movement of the skyrmion under a pulsating electric current. Thus, this research shows a way to control the properties of topological objects through the use of hybrid structures for spintronic and magnonic applications.

The paper is organized as follows. First, we analyze the skyrmion size and shape in dependence on the *D* value, and the magnetostatic fields induced by skyrmions in the isolated nanodot and the hybrid system. Next, we elucidate how the skyrmion imprint upon the stripe affects the shape of the skyrmion in the nanodot. Finally, we present a numerical proof-of-concept of the skyrmion transport in a hybrid structure composed of the skyrmion track coupled to a soft ferromagnetic stripe, which uses the specific properties of the egg-shaped skyrmion in a hybrid system to reduce the impact of the SkHE on a skyrmion transport.

## Results and discussion

We investigate a circular Co nanodot with a thickness of 1.5 nm and 150 nm radius, positioned directly 1.5 nm above a ferromagnetic Py stripe, magnetized along the positive *x*-axis (see Fig. 1). The stripe is 384 nm wide, 4.5 nm thick and 1.25 $\upmu$m long. The magnetic dot is defined with an effective medium approach as a structure^18,34–36^ with DMI and PMA, where the three repetitions of the Pt (0.5 nm)/Co (0.5 nm)/Ir (0.5 nm) multilayer stack are simulated as a single 1.5 nm layer of Co. The shape anisotropy of the stripe maintains uniform magnetization along the *x*-axis, while the assumed nonmagnetic spacer (1.5 nm thin gold layer) between the elements guarantees coupling only by the magnetostatic stray field. We perform micromagnetic simulations using Mumax3^37–39^ (details in methods section). Throughout the paper, we use the following material parameters for a nanodot: saturation magnetization $M_\text{s}$ = 956 kA/m, exchange stiffness constant $A_{\text{ex}} = 10$ pJ/m, *D*—changes in a range from 0 to 2 mJ/m$^2$, out-of-plane magnetic anisotropy constant $K_{\text{u}} = 0.717$ MJ/m$^3$. This set of parameters corresponds to the ultrathin layer of Pt/Co/Ir^1,18^ with favorable conditions for the skyrmion stabilization. For Py, we assume the following magnetic parameters: $M_\text{s} = 800$ kA/m, $A_{\text{ex}} = 13$ pJ/m^40–42^. The studied system was discretized uniformly with 0.75 $\times$ 0.75 $\times$ 1.5 nm$^{3}$ unit cells to precisely imitate the rounded geometries with high accuracy.

First, we simulate the *D*-dependent range of the skyrmion stability in the isolated nanodot (see blue dashed lines in Fig. 4). We found a stable circular skyrmion state for the *D* value from 0.86 to 1.75 mJ/m${^2}$, with skyrmion diameter increasing monotonously from 7 to 215 nm, [see, the blue dashed line in Fig. 4a,b], regardless of the *D* sign. In the range of |*D*| from 0.86 to 1.1 mJ/m$^2$ we observe a small skyrmion with a slight increase of its diameter from 7 to 35 nm. Then, the rapid increase of the diameter is observed and a large skyrmion stabilizes (skyrmion size $s_{x}>80$ nm).

**Figure 2:**
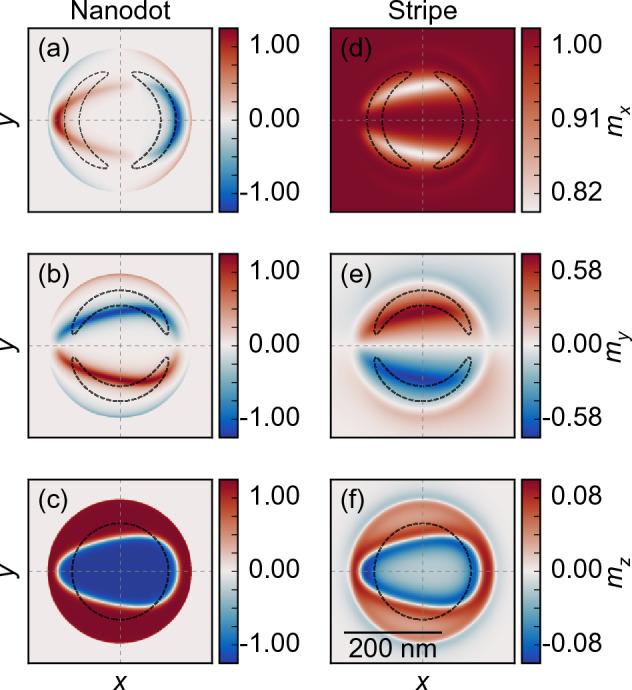
Static magnetization configuration in a Co nanodot with $D=-1.6$ mJ/m$^2$ dipolarly coupled to the Py stripe. The color scale is given in reduced units and shows components of the magnetization vector **m** of length normalized to 1. (**a**–**c**) The $m_x$, $m_y$, and $m_z$ components of magnetization in the nanodot, and (**d**–**f**) the components of the magnetization in the top surface of the stripe. The black dashed line was plotted as an isoline of 25% of the maximum positive or negative magnetization amplitude of $m_{x}$ (**a**,**b**), $m_{y}$ (**d**,**e**), and $m_{z}=0$ (**c**,**f**) of the skyrmion texture in the isolated nanodot. The central vertical and horizontal axes determine the zero point of the *x* and *y*-axes, respectively.

Next, we perform relaxation simulations of a hybrid system, a nanodot coupled with a stripe. For *D* equal to $-1.6$ mJ/m$\mathrm {^2}$ we observe strongly deformed, like an egg-shape, skyrmion [see Fig. 2a–c] with the maximum size $s_{y}= 150$ nm and $s_{x}= 240$ nm, along the *y* and *x*-axis, respectively [skyrmion size is measured as the distance between the furthest points of the skyrmion domain wall along the respective axis, where $m_{z}=0$ (see Fig. 4e)]. In Fig. 2, the black dashed lines show the contours of the skyrmion domain wall in the isolated nanodot. Note that the observed egg-shape of the skyrmion has the size along the *y*-axis on the left part of the nanodot ($x<0$) smaller than on the right part ($x>0$), which is correlated to the magnetization orientation in the stripe, as we will show in the following part.

**Figure 3:**
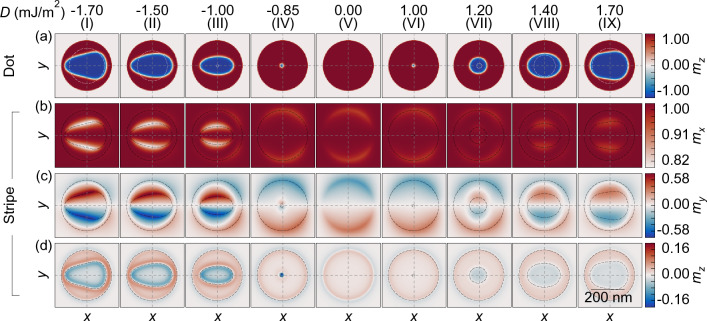
Images of the relaxed magnetization configuration of the Co nanodot over the Py stripe for different values of *D* in the nanodot. (**a**) The out-of-plane component of the magnetization in the nanodot. The white-dashed line marks the shape of the skyrmion in an isolated nanodot at the respective *D*, which is circular with the radius shown as blue dashed line in Fig. 4 (**b**, **c**). The in-plane and (**d**) the out-of-plane components of the magnetization inside the stripe-the skyrmion’s imprint. The dashed lines represent the edge of the nanodot (outer circle) and the edge of the skyrmion (inner curve).

Figure 2d–f shows the deformation of the magnetization components in the stripe from the $\textbf{m}=({m_x},0,0)$ alignment ($|m_x|=1$. The magnetization in the stripe deviated from their equilibrium position in the areas of interaction with the edge of the nanodot and the skyrmion is called an *imprint*. A larger disturbance is observed on the $m_{y}$ (up to $\pm 0.58$) than the $m_{x}$ component (up to 0.18). The reduction of the $m_{z}$ component reaches $\pm 8$% at the horizontal edges of the skyrmion. Furthermore, the magnetization components $m_{x}$ and $m_{z}$ are symmetric with respect to the $x-$axis, while $m_{y}$ demonstrates antisymmetry. With respect to the $y-$axis, the imprint exhibits asymmetry, underscoring skyrmion egg-shape nature.

**Figure 4:**
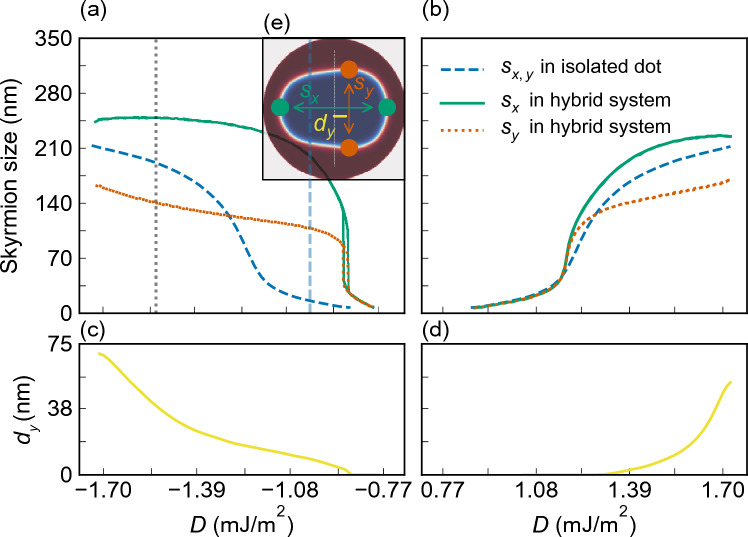
The maximal skyrmion size along the *x* and *y* axis in dependence on the *D*, (**a**) between $D =-1.75$ and $-0.75$ mJ/m$^2$ and (**b**) between $D =0.75$ and 1.75 mJ/m$^2$. The blue dashed line represents the diameter for an isotropic circular skyrmion in the isolated nanodot. Green $s_x$ and orange $s_y$ curves represent the skyrmion maximum size measured along the *x* and *y* axis, respectively, in the hybrid system. The vertical, light-blue line indicates the *D* value for which the analysis of the effect of magnetocrystalline anisotropy on skyrmion size is performed in Fig. 7. (**c**,**d**) The measure of the skyrmion asymmetry with respect to the *y*-axis, i.e., the shift of the maximum size $s_y$ of the skyrmion from the symmetry position $x=0$, $d_y$. (**e**) Image of the relaxed skyrmion for $D =1.6$ mJ/m$^{2}$, with the markings of the plotted values.

The skyrmion and the imprint dependence on the sign and strength of *D* are shown in Fig. 3. We are varying the value of *D* from $\pm 0.86$ mJ/m$^2$ (the skyrmion formation) to  $\pm 1.75$ mJ/m$^2$ (skyrmion instability). We show the $m_{z}$ component of the magnetization in the nanodot in (a), and $m_{x}$, $m_{y}$ and $m_{z}$ components in the stripe in (b–d). The most important observations are: (i) The significant increase of the skyrmion size as compared to the isolated dot (discussed in the next paragraph and drawn in Fig. 4). (ii) The stronger increase in a skyrmion size and larger egg-shape deformation at negative than positive *D* values. (iii) The magnetization deformation in the imprint is lower for positive than negative *D* (the maximal intensity of the $m_{y}$ component is about $\pm 0.59$ for the imprint at $D=-1.7$ mJ/m${^2}$, while for the positive *D* it is only $\pm 0.17$ at the normalized magnetization units). (iv) For all considered *D* values, the imprint has the same type of symmetry and asymmetry as the skyrmion. (v) The strength of the imprint induced from the edge of the nanodot becomes weaker as the skyrmion grows.

The quantitative analysis of the skyrmion change with increasing *D* for the hybrid structure and isolated dot is shown in Fig. 4. For each value of *D* presented here, the topological number of the skyrmion is preserved and is invariably around $\pm 1$. For the positive sign of *D*, up to $D =1.1$ mJ/m${^2}$, we do not observe the difference between the skyrmion size and shape of the isolated nanodot and the hybrid system, but above this value, the skyrmion in a hybrid structure deforms and increases size. Importantly, the skyrmion starts to stabilize at lower *D* values compared to the isolated dot, i.e., at $D =-0.788$ mJ/m$^2$ instead of $-0.863$ mJ/m$^2$. The sudden skyrmion growth happens also at a significantly smaller *D* magnitude in the hybrid structure than in the isolated nanodot, i.e., at $D =-0.82$ mJ/m$^2$ instead of $-1.23$ mJ/m$^2$. In addition, we also note a significant correlation between the DMI strength and the skyrmion area, as illustrated in Fig. S5. Specifically, for the entire range of negative DMI values, the skyrmion area in the hybrid system consistently surpasses that in the isolated system.

Interestingly, in a narrow range of negative *D* values, from $D =-0.82$ mJ/m$^2$ to $-0.88$ mJ/m$\mathrm {^2}$, we observe bi-stability of the skyrmion^16,18,43^, i.e., a simultaneous occurrence of two possible realizations of the skyrmion state, distinguished by different skyrmion size, i.e., 25 nm and 125 nm at $-82$ mJ/m${^2}$. For higher negative *D* values, we observe a significant disparity between the sizes of the skyrmion along the *x* and the *y* axes ($s_x$ and $s_y$, respectively), as well as a strong asymmetric deformation of the skyrmion along the *x*-axis ($d_y -$ the shift of the maximum skyrmion size along the *x*-axis from the symmetrical position), see Fig. 4a,c. This disparity reaches even 100 nm at $-1.25$  mJ/m${^2}$.

## Discussion

Although a stripe with saturated magnetization along its axis exerts a negligible stray magnetostatic field on the nanodot, we found a large difference in the shape and size of the skyrmion in the hybrid system as compared to the isolated nanodot. It is clear that the magnetostatic stray field, ${\textbf{H}}_{\text {s}}$, is responsible for these mutual interactions between the two systems and the observations described in the previous section. The stray field from the nanodot with the skyrmion ${\textbf{H}}_{\text {s-dot}}$ forms an imprint upon the stripe, which in turn induces a magnetostatic stray field ${\textbf{H}}_{\text {s-str}}$ in the nanodot. By conducting a simplified examination of the effective fields acting on individual segments of the skyrmion domain wall, and identifying the contributions arising from the imprint formed within the stripe, we can extrapolate the subsequent interaction process between the magnetic textures of the stripe (imprint) and the skyrmion. Thus, to explain these mutual interactions, we will analyze both contributions separately in the following subsections.

### Stray magnetostatic field from the nanodot

**Figure 5:**
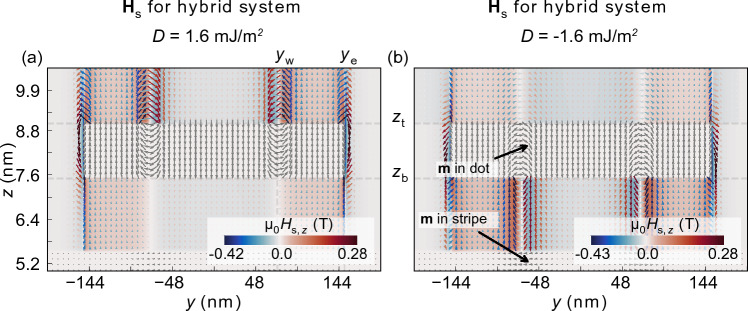
The spatial distribution of the stray magnetostatic field in the studied system, for $D=1.6$ mJ/m$\mathrm {^2}$ (**a**), and $D=-1.6$ mJ/m$\mathrm {^2}$ (**b**). The plot is taken in the (*y*, *z*) mid-plane cross-section. The central and bottom parts of the figure, marked by the white color and gray arrows represent the magnetization in the nanodot and the stripe, respectively. The color scale represents the intensity of the *z*-component of the $\textbf{H}_{\text {s}}$ magnetostatic field. The size of the arrow is proportional to the magnetic field strength.

Figure  5a presents the spatial distribution of ${\textbf{H}}_\text{s}$ in the hybrid system for $D=1.6$ mJ/m$^2$ (for this purpose, we have performed an analysis of the magnetostatic field distribution of the system under study with reduced size of the elementary cell, just to obtain precise spatial distributions of the field. We used here 0.75 $\times$0.75 $\times$0.15 nm$^{3}$ cell size). ${\textbf {H}}_{\text {s-dot}}$ is significantly larger above ($z> z _{\text {t}} = 9$ nm) than below ($z< z _{\text{b}} = 7.5$ nm) the nanodot, where $z _{\text{t}}$ and $z _{\text{b}}$ correspond to the top and bottom surface of the nanodot, respectively. We observe also the following changes in the field strength: (i) an enhancement of the fields above the skyrmion wall and the nanodot edge, (ii) a weaker but still relatively strong field below the edge of the dot, and (iii) a decrease of the stray-field value below the skyrmion wall. These properties apply to both components of the stray field, i.e., radial and vertical.

To understand the origin of this stray field asymmetry, we involve the concept of magnetostatic surface and volume charges^44,45^. With that we can distinguish three main contributions to ${\textbf{H}}_{\text {s-dot}}$: (i) ${\textbf {H}}_{\text {s-dot.e}}$ that originates from a tilted magnetization at the nanodot’s edge, (ii) ${\textbf {H}}_{\text {s-dot.dw}}$, the field induced by volume charges in a Néel-type domain wall of the skyrmion, and (iii) ${\textbf {H}}_{\text {s-dot.dm}}$, the field from the surface charges of the domains, i.e., the skyrmion core and its surrounding. The field ${\textbf {H}}_{\text {s-dot.dm}}$ has opposite radial orientation above and below the skyrmion domain wall. The bulk charges of a given chirality Néel domain wall ${\textbf {H}}_{\text {s-dot.dw}}$ have the same sign throughout the thickness, leading to a symmetric distribution of the stray field along the *z*-axis. Thus, the field enhancement or suppression is an effect of a constructive or destructive superposition of these two components, ${\textbf {H}}_{\text {s-dot}} (x_{\text {w}}, y_{\text {w}}, z_{\text {t/b}} \pm z) = {\textbf {H}}_{\text {s-dot.dw}} \pm {\textbf {H}}_{\text {s-dot.dm}}$, where $z>0$, and ($x_{\text {w}}$, $y_{\text {w}}$) indicates a domain wall lateral position. Close to the nanodot edge, the magnetization is slightly tilted from the normal direction^46^ resulting also in enhancement and suppression of the stray field intensity above and below the nanodot, respectively.

In fact, the described asymmetry in the stray field is a Halbach effect, well known in permanent magnets arrangements with the rotated magnetization but here, realized in a deep nanoscale with the chiral domain wall of the skyrmion^47,48^. Since the sign of the *D* determines the chirality of the Néel domain wall, its change reverses the areas of weakening and enhancing of the stray field. Indeed, for $D=-1.6$ mJ/m$^2$ the ${\textbf {H}}_{\text {s-dot}}$ field is enhanced below the nanodot [see Fig. 5b]. Because ${\textbf {H}}_{\text {s-dot.dw}}$, ${\textbf {H}}_{\text {s-dot.e}} < {\textbf {H}}_{\text {s-dot.dm}}$^49^, the orientation of the effective stray field from the nanodot, its radial and *z* components, is always determined by the skyrmion polarization, but its strength is controlled by the domain wall chirality, i.e., the sign of *D*.

Worth to note is that the stray field induced by the skyrmion domain wall ${\textbf {H}}_{\text {s-dot.dw}}$ reduces the effect of the stray field ${\textbf {H}}_{\text {s-dot.e}}$ induced from the dot edge as a result of the opposite rotations of these fields. Thus, as the skyrmion size increases the effective field under and above the edge of the nanodot decreases, as we have already observed in Fig. 3b,c (I–IX). Moreover, the asymmetric skyrmion deformation visible in this figure can only be related to the mutual interaction of the stripe with the stray field from the domain wall, since the edge maintains its circular symmetry. Thus, in the following two subsections, we will focus our discussion only on ${\textbf {H}}_{\text {s-dot.dw}}$ and ${\textbf {H}}_{\text {s-dot.dm}}$.

### The imprint

In Supplementary Material, Fig. S3 shows the 3D plot of the magnetization configuration in the hybrid system and the field ${\textbf {H}}_{\text {s}}$ in the space between the elements for the negative value of *D*. Below the skyrmion domain wall, the effective stray field is directed centrifugal. This field and its orientation, in particular ${\textbf {H}}_{\text {s-dot}} \approx {\textbf {H}}_{\text {s-dot.dw}} + {\textbf {H}}_{\text {s-dot.dm}}$, is important because it exerts the torque on the stripe magnetization placed below the skyrmion, causing its deformation, i.e., an imprint.

**Figure 6:**
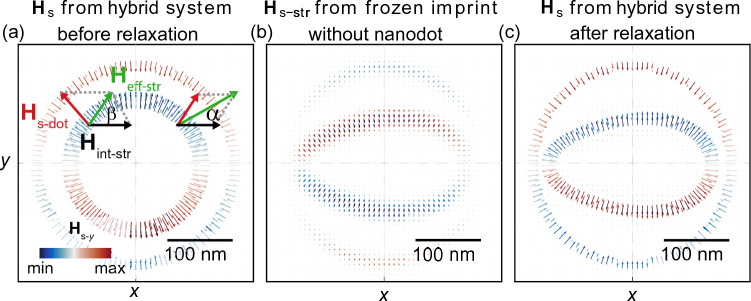
The normalized to maximum value magnetostatic stray-field distribution calculated in the space between the nanodot and the stripe ($z=6.5$ nm) for $D=1.6$ mJ/m${^2}$ generated by: (**a**) the nanodot with a circular skyrmion, (**b**) the imprint, and (**c**) in the hybrid system after relaxation. Vertical and horizontal dashed lines indicate 0 of the *x* and *y*-axes, respectively. The color scale represents the intensity of the *y*-component of the $\textbf{H}_{\text {s}}$ magnetostatic field.

Let’s consider a simplified model of the stripe and nanodot in their initial, isolated equilibrium states before the relaxation process (Fig. 6a). This approach provides us a general understanding of the system’s step-by-step relaxation process. In that case, the initial skyrmion is fully circular, i.e., it exerts a circularly symmetric stray field. Here, the effective field in the stripe **H**$_{\text {eff-str}}$ at the position of skyrmion domain wall can be considered as a sum of ${\textbf {H}}_{\text {s-dot}} ({x}_{\text {w}},{y}_{\text {w}}, z < z_{\text {b}})$ field and the internal magnetic field of the stripe, which in our case can be limited to the shape anisotropy field: ${\textbf {H}}_{\text {int-str}}=[\textrm{H}_{\text {int-str}},0,0]$. During relaxation, the magnetization in the stripe shall follow the direction of the effective magnetic field to minimize the torque. As schematically shown in Fig. 6a, the in-plane component of the effective field in the stripe ${\textbf {H}}_{\mathrm {eff-str,\parallel }}={\textbf {H}}_{\mathrm {s-dot,\parallel }}+{\textbf {H}}_{\text {int-stripe}}$ is parallel to the stripe magnetization at $y=0$ line, therefore does not exert any torque. But for $y\ne 0$ the torque is nonzero, and it has a different magnitude for the halves at $x>0$ and $x<0$. Therefore, the angle between the effective field ${\textbf {H}}_{\mathrm {eff-str,\parallel }}$ and the stripe axis is larger on the left side ($\beta > \alpha$), so the magnetization tilt from the $y=0$ line shall be stronger on the left side of the skyrmion center than on the right side. Thus, this field causes relaxation of the stripe magnetization to the asymmetric imprint texture.

It is clear that for the hybrid system after the relaxation, the in-plane components of the magnetization $m_{x}$ and $m_{y}$ in the stripe are symmetric and antisymmetric with respect to the *x*-axis, respectively, and both are asymmetric with respect to the *y*-axis.

### The asymmetric deformation of the skyrmion

Let us now, analyze how in turn, the imprint affects the skyrmion, and how the subsequent relaxation process of these mutually interacting magnetic subsystems proceeds. To get a deeper insight into the mechanism of the skyrmion deformation, we performed independent simulations for the subunit of the structure, it is a stripe with a frozen magnetic texture, i.e., a skyrmion imprint present, but a nanodot removed. Fig. 6b shows ${\textbf {H}}_{\text {s-str}} (x,y,z=6.5 \text { nm})$, i.e., the magnetostatic stray filed from the imprint in the plane between the dot and the stripe (in Supplementary Material Fig. S2 this field along the plane perpendicular to the stripe axis is shown). This stray field is opposite to and an order of magnitude lower than the field, which is created by the nanodot ${\textbf {H}}_{\mathrm {s-dot}}$ [compare Fig. 6a], with maximum stray field intensity $\upmu _{0} H_{\text {s-str}} = 0.036$ T. Comparing the imprint for positive and negative values of *D* [see Fig. 3b,c], we can see that the weaker field induced by the skyrmion for positive *D* induces a weaker imprint, which in turn generates weaker ${\textbf {H}}_{\text {s-str}}$. Moreover, as shown in the previous sub-section, the strength of the imprint, and so the ${\textbf {H}}_{\text {s-str}}$, depends also on the rigidity of the magnetization in the stripe, which is determined by its magnetic anisotropy. This clearly indicates that the amplitude of ${\textbf {H}}_{\text {s-str}}$ field is proportional to the local deformation of the imprint. Thus, the larger imprint, the stronger *y*-component field at the skyrmion-domain wall position, and it is oriented toward the *x*-axis [see, Fig. 6b]. This stray-field component influences the skyrmion resulting in its squeezing along the *y*-axis, and finally deformation of the circular skyrmion to the elliptical shape [see, Fig. 6c]. The stronger ${\textbf {H}}_{\text {s-str}}$ field for $x<0$, then at $x>0$ (under assumed geometry), additionally deforms the ellipsoidal shape towards the egg-shape skyrmion as seen in Figs. 2 and 3, with the side of larger deformation determined by the magnetization orientation in the stripe.

The last unexplained effect observed in simulations in Figs. 3 and 4 is a significant enlarging of the skyrmion size in the hybrid structure with respect to the isolated dot, above *D* value $-0.88$ and 1.16 mJ/m$^2$, for negative and positive *D*, respectively (see also Fig. S5). We attribute this effect to the enhancement of the internal magnetic field, in particular, its *z* component, inside the nanodot by the out-of-plane component of the imprint magnetization, which is always parallel to the polarization of the nanodot domains, see Fig. S4 in Supplemental Material. Thus, the influence of the surface charges from the imprint can be similar to the role of the external magnetic field parallel to the skyrmion core.

**Figure 7:**
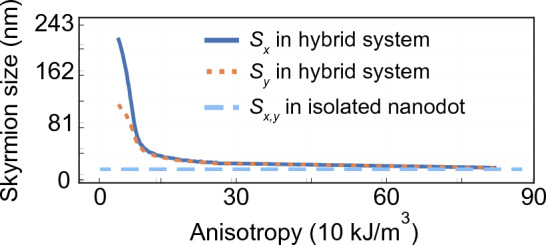
The skyrmion size (its maximal dimensions along the *x* and *y* axis) in dependence on the magnetic anisotropy constant of the stripe. In the calculations, we assumed the $D=-1.0$ mJ/m$^{2}$. The skyrmion size indicated with a blue dashed line corresponds to a 20 nm diameter skyrmion found in an isolated dot.

Our discussion focuses on the analysis of magnetostatic fields for different values of *D*, but the degree of deformation is also affected by the strength of exchange interactions or effective anisotropy in the nanodot as well as in the stripe. To demonstrate the impact of the stripe’s internal magnetic field on the nanodot’s skyrmion shape, we performed simulations in which we assume uniaxial (parallel to the *x* axis) magnetic anisotropy in the Py stripe with anisotropy constant $K_{\text {u}}$. Figure 7 presents simulation results for a hybrid system at $D=-1.0$ mJ/m$^{2}$, where the increase of $K_{\text {u}}$ clearly results in the decrease of skyrmion size and the skyrmion asymmetry. Starting with $K_{\text {u}} = 0$ [the egg-shaped skyrmion of 200 nm maximal size along the *x*-direction, see vertical light-blue dashed line in Fig. 4a] the skyrmion size and its asymmetry decrease with increasing anisotropy up to $K_{\text {u}} \approx 10$ kJ/m$^3$, above it reaching a circular shape. Further increase in anisotropy constant results in a slow monotonous reduction of the skyrmion size, and for $K_{\text {u}} = 30$ kJ/m$^3$ the skyrmion has only 25 nm in diameter, close to the size of a skyrmion in the isolated nanodot, 20 nm. Thus, by controlling the strength of the internal magnetic field in the stripe, e.g., via uniaxial anisotropy (shape, magnetocrystalline, or strain induced) we can control the degree of deformation and the size of the skyrmion, and so control the effective role of the *D* in the nanodot.

**Figure 8:**
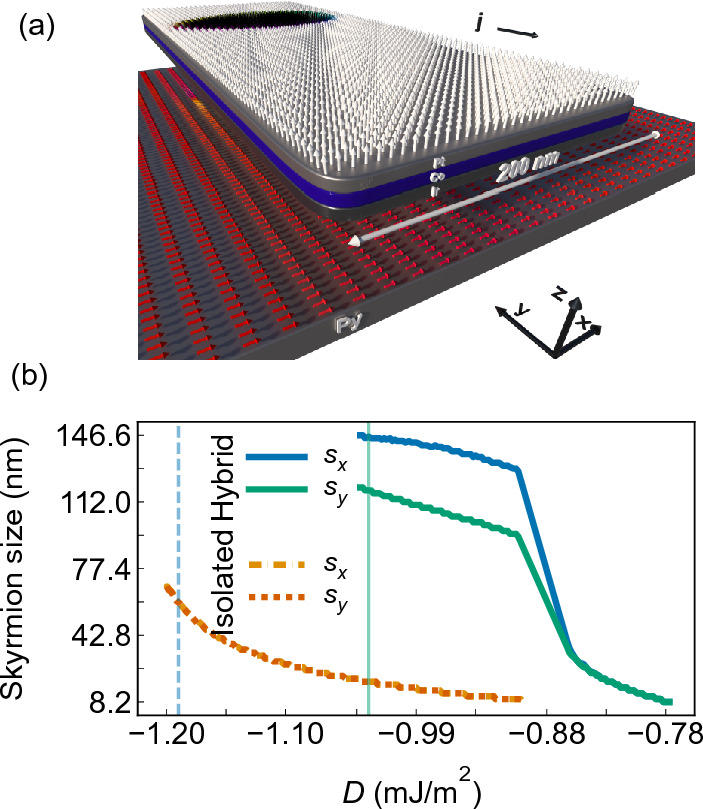
(**a**) Schematic illustration of the investigated hybrid-racetrack. Note that the figure is not to scale. The 200 nm wide multilayer Pt/Co/Ir racetrack is separated by a 1.5 nm thick Au layer from Py stripe. The arrows and their color (according to the HSL cone color scale) indicate the direction of magnetization. (**b**) The skyrmion size dependence on *D* strength for the isolated (orange and red dashed line) stripe and hybrid racetrack (blue and green solid lines).

### Application outlook

In the last section, we examine the potential application of a hybrid system for racetrack memory. Specifically, we will demonstrate that the unique properties of our system can mitigate the SkHE. We conduct micromagnetic simulations of 200 nm wide and 1.5 nm thick Co stripe, which effectively represents the Pt/Co/Ir multilayer stack [shown in Fig. 8a] playing a role of the racetrack, separated by a 1.5 nm Au layer from Py layer of 4.5 nm thickness and 1.25 ${\upmu }$m width. To preserve the in-plane magnetization of the Py layer perpendicular to the Pt/Co/Ir stripe axis, we add uniaxial anisotropy of $K_{\text {u}}=3.5$ kJ/m$^{3}$.

As the geometry of the skyrmion host layer has changed, we repeat the study of the skyrmion stability in dependence on *D* for the hybrid racetrack and isolated Pt/Co/Ir stripe. The results are shown in Fig. 8b. The presence of a Py layer near the racetrack changes the range of skyrmion stability toward smaller *D* values and significantly increases their size (green and blue solid lines) in comparison to the isolated racetrack (orange dashed line). For an isolated Pt/Co/Ir racetrack, we found circular Néel type skyrmion for *D* in the range from $-0.89$ mJ/m$^{2}$ to $-1.20$ mJ/m$^{2}$, where the skyrmion diameter varies from 8.2 to 76.5 nm, i.e., small skyrmions. For larger *D* values, the skyrmion begins to grow anisotropically toward the long axis of the racetrack and eventually evolves into a complex magnetic texture. In the hybrid racetrack, we obtain a stable skyrmion already at $D=-0.78$ mJ/m$^{2}$ with a circular shape and diameter 8.2 nm. When the *D* value reaches $-0.84$ mJ/m$^{2}$, it initiates a rapid and anisotropic expansion of its size. We found that the skyrmion size varies within a range of 110–119 nm along the *x*-axis and 130–146.2 nm along the *y*-axis in the *D* range between $-0.90$ and $-1.03$ mJ/m$^2$ (see a blue and green solid line). This shows that the width of the racetrack limits the maximum size of the skyrmion along the $x-$axis, while magnetostatic interactions with the imprint limit the skyrmion’s growth along the racetrack. At $D<-1.03$ mJ/m$^2$ and $D<-1.20$ mJ/m$^2$ for the hybrid and isolated racetrack, respectively, irregular domains filling the entire space of the racetrack are formed.

**Figure 9:**
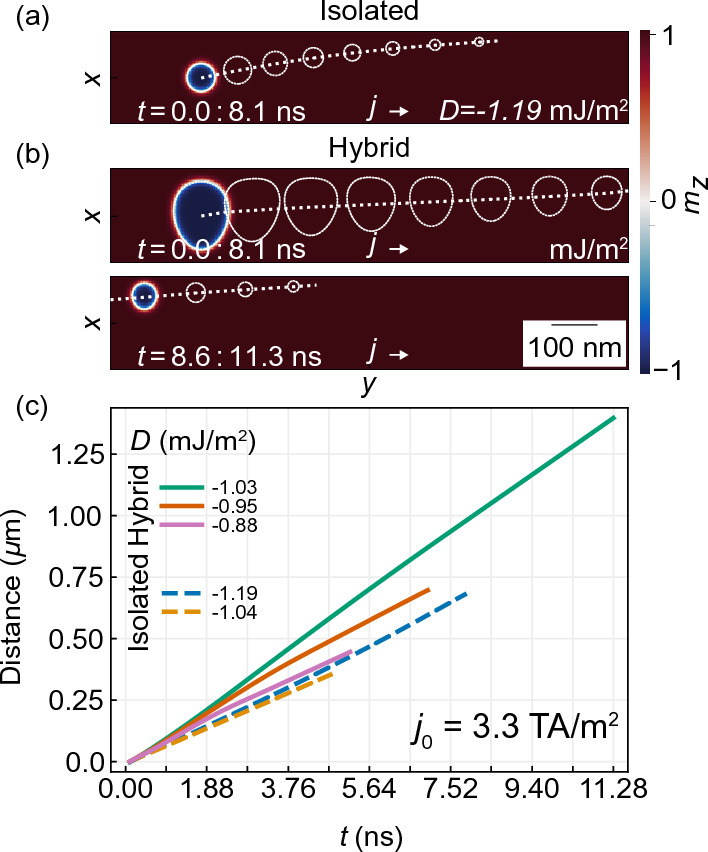
(**a**,**b**) Simulated illustration of skyrmion moving trajectory driven by continuous electric current in isolated (**a**) and hybrid (**b**) racetrack. Panel (**b**) was divided into two sections to present the full skyrmion trajectory from the initial point to annihilation. White-dashed contours present a skyrmion position in every 1 ns steps. The straight dashed line presents the skyrmion trajectory. (**c**) Location of a skyrmion driven by a continuous electric current along the racetrack for different values of *D*. Dashed and solid lines are for the isolated and hybrid racetracks, respectively. Regardless of the system and *D* value, skyrmion annihilates as a result of interaction with the edges of the racetrack resulting from SkHE.

Next, we conduct micromagnetic simulations to study the motion of skyrmion driven by a spin-polarized electron direct current (with density $j_{0} = 3.3$ TA/m$^2$) applied along the $y-$axis and limited to the Pt/Co/Ir multilayer region, see Fig. 9 (simulations details are in the method’s section). In Fig. 9a,b, the white dashed lines show the trajectory of the skyrmion, while the white skyrmion contours present the following skyrmion positions with a time interval of 1.0 ns for the isolated ($D=-1.19$ mJ/m$^2$) and hybrid ($D=-1.03$ mJ/m$^2$) racetrack, respectively. In both cases, the topological skyrmion charge leads to the emergence of a gyroscopic force that causes a deflection of the skyrmion towards the racetrack edge. The simulations results of skyrmion position along the racetrack as a function of time for $D=-0.88,-0.95,-1.03$ mJ/m$^{2}$, and $D=-1.04$ mJ/m$^2$ and $-1.19$ mJ/m$^{2}$ for a hybrid and isolated racetrack, respectively, are presented in Fig. 9c. They show that when a continuous spin-polarized electric current with current density $j_{0}=3.3$ TA/m$^2$ is applied, the skyrmion in both the isolated and hybrid racetrack moves, but the distance between the skyrmion and the edge of the racetrack decreases, and eventually the skyrmion is completely annihilated^50,51^. We found that the skyrmion’s maximum propagation distance, till annihilation with the racetrack edge, correlates with the size of the skyrmion^52^. For the isolated racetrack, the skyrmion maximum propagation distance is $0.684~\upmu$m, and $0.35~\upmu$m at $D=-1.19$ mJ/m$^2$ and $D=-1.04$ mJ/m$^2$ (as shown by the blue and orange dashed lines), where we found $s_{xy}=60$ nm and 20.7 nm skyrmion diameter, before applying electric current, respectively. For the hybrid racetrack, for the comparable *D* values, i.e., at $D=-1.03$ mJ/m$^2$, the maximum distance was 67% longer in comparison to skyrmion in the isolated racetrack. The skyrmion velocity in the isolated racetrack is similar for both large and small skyrmions, it’s around 89 m/s. The propagation velocity of a skyrmion in a hybrid structure increases with increasing their size, for example, for $D=-0.88$ mJ/m$^2$ (initial skyrmion size $s_x=74.5$ nm, and $s_y=60.7$ nm) it was similar to the isolated racetrack, but it increases to 123 m/s at $D = -1.03$ mJ/m$^2$ (initial skyrmion size $s_y=144.9$ nm, and $s_y=118.6$ nm). This points out that skyrmions in a hybrid racetrack can propagate farther and faster for a given *D* than in an isolated racetrack.

We found that, by interrupting the current flow, the skyrmion in the hybrid racetrack rapidly grows and may return to its original size, and shape, filling the entire width of the waveguide, and maintaining its position along the waveguide. This observation allows us to propose a scenario for skyrmion transport along the long track by a sequence of current pulses^50,53^, with a short relaxation time between them.

**Figure 10:**
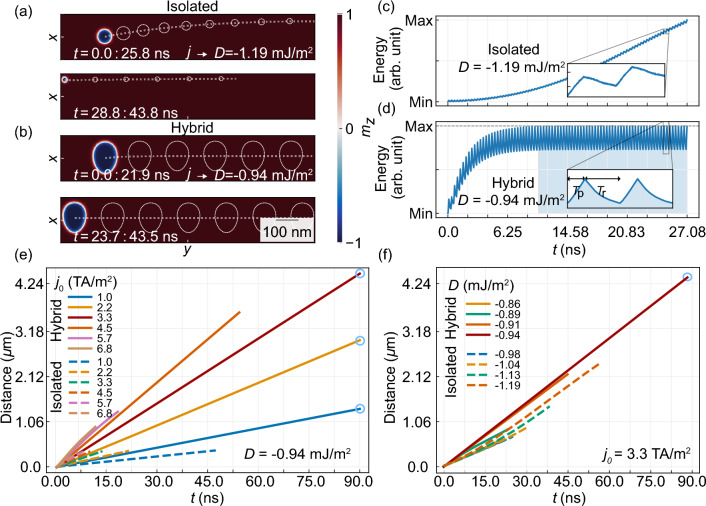
(**a**,**b**) Illustration of skyrmion moving trajectory driven by a sequence of electric current pulses in isolated (**a**) and hybrid (**b**) racetrack, respectively. Panels (**a** and **b**) were divided into two sections to present the full skyrmion trajectory from the initial point to annihilation (**a**) and further in time evolution (**b**), respectively. The current pulse sequence was constructed with a 0.1 ns electric current pulse ($T_{\textrm{p}}$) of density $j_{0}=3.3$ TA/m$^2$ with the following 0.2 ns of relaxation ($T_{\text {r}}$). White-dashed contours present the skyrmion position every 3.3 ns. The straight dashed line presents the skyrmion trajectory. Panels (**c**,**d**) present the total energy dependence on time with current density $j_{0}=3.3$ TA/m$^{2}$ for the hybrid (**d**) and isolated (**c**) racetracks. (**e**,**f**) Distance versus time for the skyrmion driven by a sequence of electric current pulses for different values of *D* (**f**) and current densities $j_{0}$ (**e**). Dashed and solid lines have been plotted for an isolated and hybrid racetrack, respectively. The blue circle indicates that in the simulated time, the skyrmion has not annihilated. Current dependent simulations were performed for $D=-0.94$ mJ/m$^2$.

To verify our concept, we applied the electric current along the *y* axis in a form of a time-dependent square function:

1 $$\begin{aligned} j_{y} (t) = {\left\{ \begin{array}{ll} \textit{j}_{0} &{} \text {if } t \in (0 ; T _{\textrm{p}}) ,\\ 0 &{} \text {if } t \in ( T _{\textrm{p}}; T _{\textrm{r}}) , \end{array}\right. }, \end{aligned}$$

where $j_{0}$ is a current density amplitude 3.3 TA/m$^2$, $T _{\textrm{p}}$ is a time of applied current forming together with the relaxation time $T _{\textrm{r}}$ a sequence period ($\textit{T}= T _{\textrm{p}}+ T _{\textrm{r}}$). Fig. 10a,b shows a simulated skyrmion moving trajectory driven by a sequence of electric current pulses in isolated (a) and hybrid (b) racetracks, respectively. Here, pulses of electric current ($T_{\text {p}}=0.1$ ns) are followed by full relaxation ($T_{\text {r}}=0.2$ ns) processes. The skyrmion movement is shown by the white skyrmion domain wall contours with a time interval of 3.3 ns between neighboring contours. The white dashed lines show the trajectory of the skyrmion motion in this sequence. The timestamp of the sequential movement of the skyrmion is of a stepped pattern, consisting of moving (during $T_{\textrm{p}}$) and relaxation (during $T_{\text {r}}$) periods. Here, similarly to the continuous flow of spin current, skyrmions due to the SkHE are driven out of their equilibrium position during $T_{\text {p}}$, deforming it, and increasing the total energy. However, during $T_{\text {r}}$ stages, the skyrmion partially restores its initial shape and energy, but differently for the isolated and hybrid racetrack. Thus, to analyze the skyrmion mobility in the isolated racetrack at $D=-1.19$ mJ/m$^2$ and hybrid racetrack at $D=-0.94$ mJ/m$^{2}$ as shown in Fig. 10a,b, we will discuss the evolution of their total energy shown in Fig. 10c,d, respectively.

In the isolated racetrack, Fig. 10c, skyrmion during each $T_{\text {p}}$ stage underwent deformation and shrinkage, resulting in an increase in the total energy of the system. During $T_{\text {r}}$ stages skyrmion reduces energy and restores its shape, however, doesn’t return to the center of the racetrack. Thus, the assumed relaxation time does not allow the skyrmion to return to its original shape and position, so with further repetitions of the current pulses the skyrmion approaches the edge of the racetrack, leading to its annihilation, as indicated by the continuous increase of total energy [see Fig.  10c]^50,54,55^.

In a hybrid racetrack shown in Fig. 10b,d, we found that the skyrmion does not annihilate, as its vertical deflection is fully compensated during the relaxation process. Here, the total energy accumulation visible in first 25 periods (Fig. 10d) indirectly expresses the degree of deformation and breakout from the equilibrium position of the skyrmion (after this period skyrmion is shifted up 20 nm along *y*-axis, and is 8 nm wider in *x*-axis). In the same time the energy dissipation during the $\textit{T}_{\text {r}}$ stages increases. At each relaxation stages in hybrid system, skyrmion partially restores the original dimensions and position across the racetrack width, faster than the skyrmion in an isolated racetrack [compare the energy change during $\textit{T}_{\text {r}}$ stages in Fig. 10c,d]. This is because, the repulsion from the edge, combined with the skyrmion’s tendency to fill the entire width of the racetrack due to magnetostatic interactions with the imprint, led to faster energy dissipation in each further relaxation stages. Consequently, after 7.5 ns of sequential movement (25 periods), the skyrmion reaches a steady state by balancing the energy gain and dissipation, as indicated by flat envelope of the total energy as a function of time [see Fig.  10d]. This remains constant for each further sequence period and preserves the skyrmion’s position across the stripe. The amount of energy reduction during the $\textit{T}_{\textrm{r}}$ phase is 36% higher relative to the energy difference for isolated racetrack [see Fig. 10c,d].

In Fig. 10e,f we evaluate the impact of the spin-polarized electric current density and *D* value, respectively, on the maximum distance traveled by skyrmions in both hybrid and isolated racetracks. For both hybrid and isolated racetracks we have shown the current position of the skyrmion at the end of each current pulse period, without plotting the skyrmion position during $\textit{T}_{\text {r}}$ phases. The simulations revealed that for the isolated racetrack, an increase in the current density results in an increase of skyrmion effective velocity, however, has no significant effect on the maximum traveled distance by the skyrmion. For example, when the current density increases from 1.0 to 6.6 TA/m$^{2}$, the maximum distance reduces from 0.36 to 0.275 $\upmu$m. This is because the relaxation period does not significantly affect the shape and position of the skyrmion, moreover, the larger amplitude of the current pushes the skyrmion faster to the racetrack edge. The skyrmion behavior in the hybrid racetrack is different. An increase in the current density increases propagation distance, and already $j_{0}=3.3$ TA/m$^{2}$ at $D=-0.94$ mJ/m$^2$ allows for infinite skyrmion propagation. Importantly, an increase of $j_{0}$ also significantly increases the skyrmion’s average velocity from 15 to 48 m/s with increasing current density from 1 to 3.3 TA/m$^2$. However, current density $j_{0}=3.3$ TA/m$^{2}$ was found to be a critical value above which skyrmion annihilates due to too short relaxation time.

In Fig. 10f we show the skyrmion maximum propagation distance at $\textit{j}_{0}=3.3$ TA/m$^2$, in dependence on *D* values in the range from − 0.86 mJ/m$^{2}$ to -0.94 mJ/m$^{2}$ and from $-0.98$ to $-1.19$ mJ/m$^{2}$ for hybrid and isolated racetrack, respectively. For the isolated racetrack, the maximum distance of skyrmion propagation decreases as the value of *D* decreases. For example, for $D=-1.19$ mJ/m$^{2}$ and $D=-0.98$ mJ/m$^{2}$ the maximum propagation distance is 2.28 $\upmu$m and 1.2 $\upmu$m, respectively. Here, the averaged skyrmion velocity varies from 25.27 to 38.28 m/s. We found also, that the sequence of driving force and relaxation increases the maximum distance almost twice when compared to a continuous current for all *D* values [as seen in the skyrmion trajectory in Figs. 9a and 10a].

For a hybrid racetrack, the maximum propagation distance is larger for comparable *D* values, e.g., for $D=-0.86$ mJ/m$^{2}$ and $D=-0.91$ mJ/m$^{2}$ the maximum propagation distance is 0.6 $\upmu$m and 2.06 $\upmu$m, respectively. For *D* smaller than $-0.94$ mJ/m$^{2}$, we don’t, observe skyrmion annihilation in the simulated time (90 ns). It is noteworthy that the average velocity of the skyrmion is uniform and weakly dependent on the *D* value in the range from $-0.94$ to $-1.03$ mJ/m$^2$ and shows infinite propagation in the simulated time (to increase the readability of the figure, we have plotted the line only for $D=-0.94$ mJ/m$^2$). Above this *D* value, as a result of the balance between magnetostatic fields and the SkHE, the initial skyrmion size, its shape, and size at the steady state are almost equal.

Using electric current pulses with an available experimentally range of density up to few TA/m$^{2}$, skyrmion can reach the typical velocity of 10–100 m/s^56,57^, and in hybrid structures it can reach a velocity even up to 750 m/s with a current density of 5.0 TA/m$^2$, see Wu et al. in Ref.^54^. However, with strong SkHE and short propagation distance. In our paper, we demonstrate the unconstrained movement of a skyrmion along the hybrid racetrack and show that the skyrmion velocity reaches 48 m/s and can be further optimized. Proposed hybrid racetracks allow the stabilization of large skyrmions at lower *D* which opens up opportunities for further geometric and material optimization. In order to obtain high-velocity, the crucial is establishing the duration of the electric current pulse relative to the relaxation time, which depends on material parameters, damping, and temperature, in addition to an electric current density. However, such studies need separate consideration.

## Conclusions

Using micromagnetic simulations we described the skyrmion’s symmetry-breaking magnetostatic interactions in the system composed of the nanodot, possessing a Néel skyrmion and the soft ferromagnetic stripe. In this system, the skyrmion exerts a stray magnetostatic field that deforms the magnetization in the stripe. Consequently, the magnetization texture imprinted upon the stripe exerts a stray field on the skyrmion, causing its enlarging and deforming into an egg-like shape. We found that in this hybrid system, the size of the skyrmion is much more sensitive to the changes in the *D* value than in the isolated dot. We demonstrated that here the *D* range of skyrmion stability is extended, allowing skyrmion stabilization at relatively low *D* values. Additionally, we found that for some range of *D* values, the interaction with the stripe creates suitable conditions for bi-stability of the skyrmion state, corresponding to a large and a small skyrmion, it is with the size above 110 nm and below 40 nm, respectively.

We show that the skyrmion and its deformation in hybrid structures of this type can be controlled by internal and external parameters (see graphical summary in Fig. S4, Suppl. Mater.). In particular, the polarization of the skyrmion core does not affect the strength of the interactions between the skyrmion and the stripe, but it controls the asymmetry orientation of the skyrmion with respect to the plane perpendicular to the axis of the stripe. Analogous changes can be achieved by reversing the orientation of the magnetization in the stripe. The sign of the *D* determines the chirality of the Néel skyrmion and so the side (above or below) of the nanodot with the enhanced stray magnetostatic field. While direct control of DMI is challenging, it can be effectively managed by appropriate sample fabrication or employing sophisticated experimental methods^58–60^. This Halbach effect on the nanoscale can be used to increase or decrease the mutual interaction of the nanodot with the stripe, by the change of the order deposition in the multilayer. Finally, the skyrmion size and deformation strength can be controlled by the internal magnetic field of the stripe, in particular by the effective magnetic anisotropy or bias magnetic field.

The hybrid systems can be used to overcome the skyrmion Hall effect undesirable in the application context and is suitable to transport a skyrmion along the straight track by electric current pulses. We have demonstrated technique for the unconstrained transport of skyrmions along a hybrid racetrack composed of a typical racetrack magnetostatically coupled with an in-plane magnetized soft ferromagnetic layer. Thus, we show numerically that the magnetostatic interactions in chiral 3D structures can be utilized for the design of better skyrmionic and magnonic devices.

## Methods

The micromagnetic simulations are performed by using the Mumax^39^ which solves the Landau-Lifshitz-Gilbert equation:

2 $$\begin{aligned} \frac{\text {d}\textbf{m}}{\textrm{d}t}=\gamma \upmu _0 \frac{1}{1+\alpha ^{2}} (\textbf{m} \times \textbf{H}_{\textrm{eff}}) + \alpha \upmu _0 \left( \textbf{m} \times (\textbf{m} \times \textbf{H}_{\textrm{eff}}) \right) , \end{aligned}$$

where ${\textbf {m}} = {\textbf {M}} / M _{\textrm{s}}$ is the normalized magnetization, ${\textbf {\text {H}}}_{\textrm{eff}}$ is the effective magnetic field acting on the magnetization, $\gamma =-1.7595 \cdot 10^{11}$ Hz/T is the gyromagnetic ration, $\alpha$ is the Gilbert damping. In this paper, the following components were considered for the effective field ${\textbf {H}}_{\textrm{eff}}$: demagnetizing field ${\textbf {\text {H}}}_{\textrm{d}}$, exchange field ${\textbf {\text {H}}}_{\textrm{ex}}$, Dzyaloshinskii–Moriya exchange field ${\textbf {\text {H}}}_{D}$, and uniaxial anisotropy field ${\textbf {\text {H}}}_{\textrm{Ku}}$. External magnetic field and thermal effects were neglected. Thus, the effective field ${\textbf {H}}_{\textrm{eff}}$ is expressed as:

3 $$\begin{aligned} {\textbf {H}}_{\textrm{eff}} = {\textbf {H}}_{\textrm{d}} + {\textbf {H}}_{\textrm{ex}} + {\textbf {H}}_{\textrm{D}} + {\textbf {H}}_{\textrm{Ku}}, \end{aligned}$$

where

4 $$\begin{aligned} {\textbf {H}}_{\textrm{ex}} = 2 \frac{A_{\textrm{ex}}}{\upmu _0 M _{\textrm{s}}} \Delta {\textbf {m}}, \end{aligned}$$

where $A_{\textrm{ex}}$ is the exchange constant, and

5 $$\begin{aligned} {\textbf {H}}_{\textrm{D}} = \frac{2 D }{\upmu _0 M _{\textrm{s}}} \left( \frac{\partial m_z}{\partial x},\frac{\partial m_z}{\partial y},-\frac{\partial m_x}{\partial x},-\frac{\partial m_y}{\partial y}, \right) . \end{aligned}$$

The uniaxial anisotropy is in the form:

6 $$\begin{aligned} {\textbf {H}}_{\textrm{Ku}} = \frac{2\textit{K}_{\textrm{u}}}{\upmu _0 M _{\textrm{s}}} \left( {\textbf {u}} \cdot {\textbf {m}} \right) {\textbf {u}}, \end{aligned}$$

where $\textit{K}_{\textrm{u}}$ is the first order uniaxial anisotropy constant and ${\textbf {u}}$ is a unit vector indicating the anisotropy direction.

The initial state in the micromagnetic simulations was assumed to be a small-diameter skyrmion state in the nanodot, and a uniformly magnetized state in the lower layer with a slight deviation from the long axis of the strip (to counteract the supersymmetry effect in micromagnetic simulations). The relaxation simulations of the hybridized system were performed using a sequence of Mumax built-in relaxation functions in four steps: minimization (*minimize*), relaxation (*relax*) with Gilbert damping $\alpha =1.0$ (for Co and Py) and default energy threshold values, solving the full LLG equation (simulation run for 1 ns), and energy minimization again. This sequence allowed us to obtain stable magnetization configuration. In all simulations for skyrmion relaxation, we assume the Néel-type skyrmion as the initial magnetization configuration. The bi-stable skyrmion states were computed by performing simulations of skyrmion stabilization as a function of *D* intensity, assuming the two skyrmion relaxations, one when the initial state is a large-diameter skyrmion (75% of the disk diameter) and the second with a small-diameter skyrmion (10% of the disk diameter).

We used the frozen-spin technique (using a *frozen* function of Mumax) to extract the spatial distribution of the magnetostatic fields induced only from a deformed skyrmion or an imprint, without the presence of a second magnetic element (see Fig. 6b and Fig. S2). We calculated these distributions by freezing the magnetic texture after relaxation and simultaneously removing the second element, then by performing one simulation step, the solver automatically calculates the magnetostatic field distributions. The sum of field distributions from both elements is equal to the field intensity obtained for the hybrid system.

In order to model the influence of the uniform electric current density on the skyrmion we use the Zhang-Li extension to the LLG equation^38,61^:

$$\begin{aligned} \frac{\text {d}\textbf{M}}{\textrm{d}t}=&\gamma \upmu _0 \frac{1}{1+\alpha ^{2}} (\textbf{m} \times \textbf{H}_{\textrm{eff}}) + \alpha \upmu _0 \left( \textbf{m} \times (\textbf{m} \times \textbf{H}_{\textrm{eff}}) \right) \\&- \frac{\nu }{ M ^{2}_{\textrm{s}}}\textbf{m} \times \left( \textbf{m} \times \hat{j} \cdot \nabla {\textbf {m}} \right) - \frac{\xi \nu }{ M _{\textrm{s}}}\left( \textbf{m} \times \hat{j} \cdot \nabla {\textbf {m}} \right) , \end{aligned}$$

The current density appears only throughout the quantity $\nu$, which is defined as:

7 $$\begin{aligned} \nu = \frac{P j_{0} \upmu _{B}}{ e \textit{M}_{\textrm{s}} (1+{\xi ^{2}})}, \end{aligned}$$

where $\hat{j}$ is the unit vector defining the direction of the electric current flow, *P* is the degree of polarization of the spin current, $\xi$ is the degree of nonadiabaticity, $\upmu _{B}$ is Bohr magneton, *e* is the electron charge.

We perform micromagnetic simulations based on the electric current parameters found in the literature for the Pt/Co multilayer structure^1,18,62,63^. However, we assumed a Gilbert damping $\alpha =0.1$ for cobalt layer and $\alpha =0.01$ for Py layer, and high intensities of electric current ($j_{0}>1$ TA/m) to intentionally increase the skyrmion Hall angle, and thus shorten the maximum propagation distance of the skyrmion, leading to its annihilation at the edge of the racetrack. These changes were necessary to demonstrate the properties of unconstrained transport of hybrid skyrmions due to the computational complexity of the numerical simulations and to shorten the calculation time. We used positive polarization $P=0.4$ and parameter $\xi =0.2$, we also assumed the uniform spatial distribution of the current density^62,63^.

For racetrack simulations we used Runge-Kutta RK45 solver with the following time-step parameters $\texttt {maxerr}=1\cdot 10^{-5}$, mindt =$1.0\cdot 10^{-14}$, maxdt = $3.0\cdot 10^{-14}$. We assumed 32 repetitions along the *x*-axis and one along the *y*-axis in the periodic boundary conditions. These simulations were performed for Py rectangular stripe of 1200 $\times$ 384 $\times$ 4.5 nm$^3$ using a uniformly discretized grid with a cell size of 0.75 $\times$ 0.75 $\times$ 1.5 nm$^{3}$ along the *x*, *y*, and *z* axes, respectively. The width of the racetrack is 300 nm and its length is 975 nm.

### Supplementary Information

Supplementary Figures.

## Data Availability

All the data supporting the findings of this study are available from the corresponding authors upon reasonable request.

## Code availability

The codes used to generate the data of this study are available from the corresponding authors upon reasonable request. The figure was prepared using Blender version 3.6 LTS (https://www.blender.org), and 3ds Max 2022 (https://www.autodesk.com/products/3ds-max).
