# Supplementary material for: Stabilization and racetrack application of asymmetric Néel skyrmions in hybrid nanostructures
Source: Sci Rep. 2023 Aug 21;13:13572. doi: 10.1038/s41598-023-40236-z (PMC10442414; doi:10.1038/s41598-023-40236-z)
Supplement: Supplementary file 1 — Supplementary Figures. [file 41598_2023_40236_MOESM1_ESM.pdf]

Supplemental Material

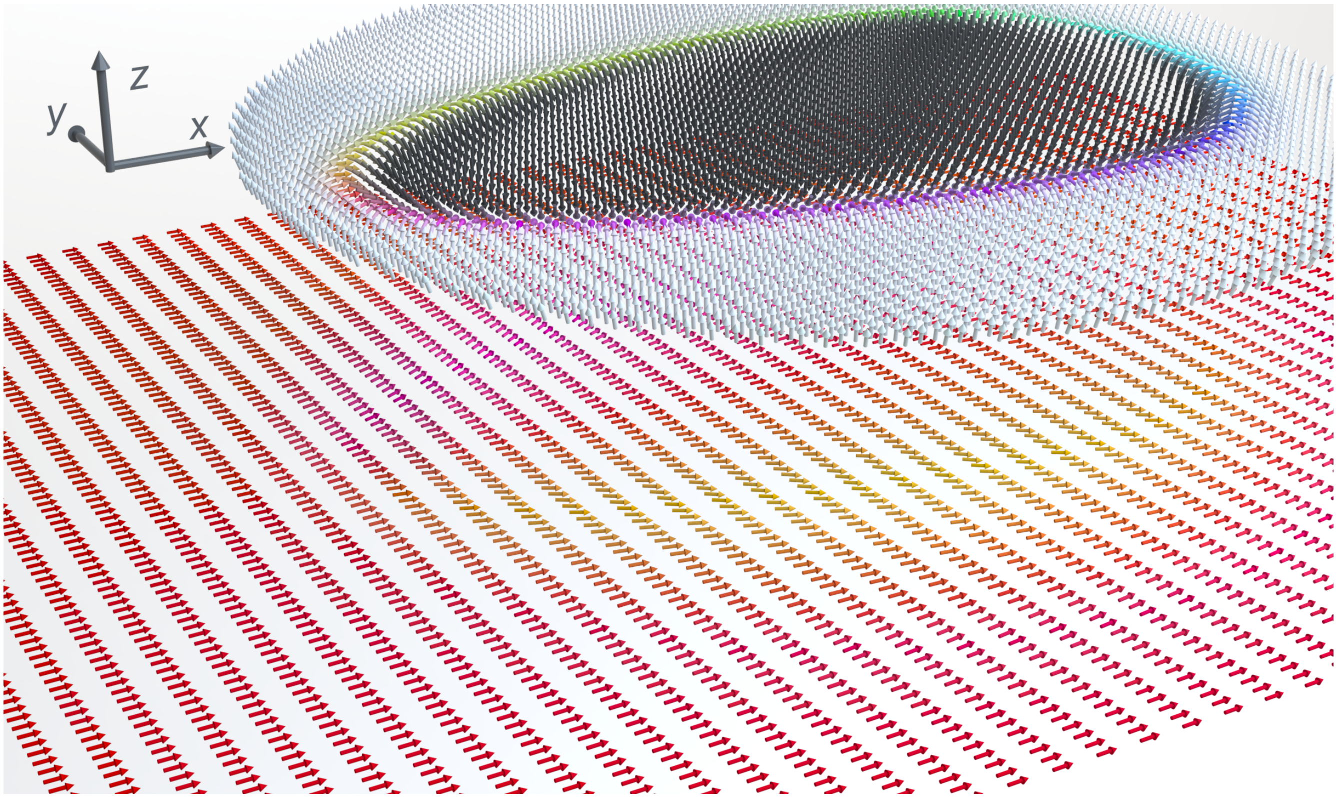

Figure S1: An artistic, high-resolution visualization of the simulated three-dimensional spatial distribution of the magnetic moments in nanodot and ferromagnetic stripe.

To demonstrate the spatial distribution of the magnetic texture magnetization of Néel-type nanodot and the imprint in the stripe, a simulated high-resolution magnetic 3D spatial distribution of the magnetisation texture is shown in Fig. S1.

Fig. S2 presets a simulated high-resolution magnetic 3D spatial distribution of the magnetization texture and $\mathbf{H}_{\mathrm{s-str}}(x,y,z=6.5\ \mathrm{nm})$, the magnetostatic stray filed from the imprint. This stray field is opposite to and an order of magnitude lower than the field, which is created by the nanodot $\mathbf{H}_{\mathrm{s-dot}}$ S3.

To understand the effects of skyrmion core polarization, D sign, and stripe magnetization direction on skyrmion shape and size, we performed a series of simulations for $D=-1.6\ \mathrm{mJ/m^2}$ of hybrid nanostructure (see Fig. S4). Micromagnetic simulations showed that a horizontal change in the direction of magnetization orientation in the stripe changes the side of the narrower side of the skyrmion. Also an alteration in the skyrmion polarity in the stripe results in a horizontal mirror image of the skyrmion's magnetization texture in the nanodot.

As depicted in Fig. S5, we observe a distinct relationship between the skyrmion area and the DMI value in both the hybrid and isolated systems. The solid orange line represents the skyrmion area in the hybrid system, while the dashed blue line corresponds to the isolated system. Interestingly, across the entire range of negative DMI values, the skyrmion area in the hybrid system consistently exceeds that in the isolated system. This observation underscores the influence of the system configuration

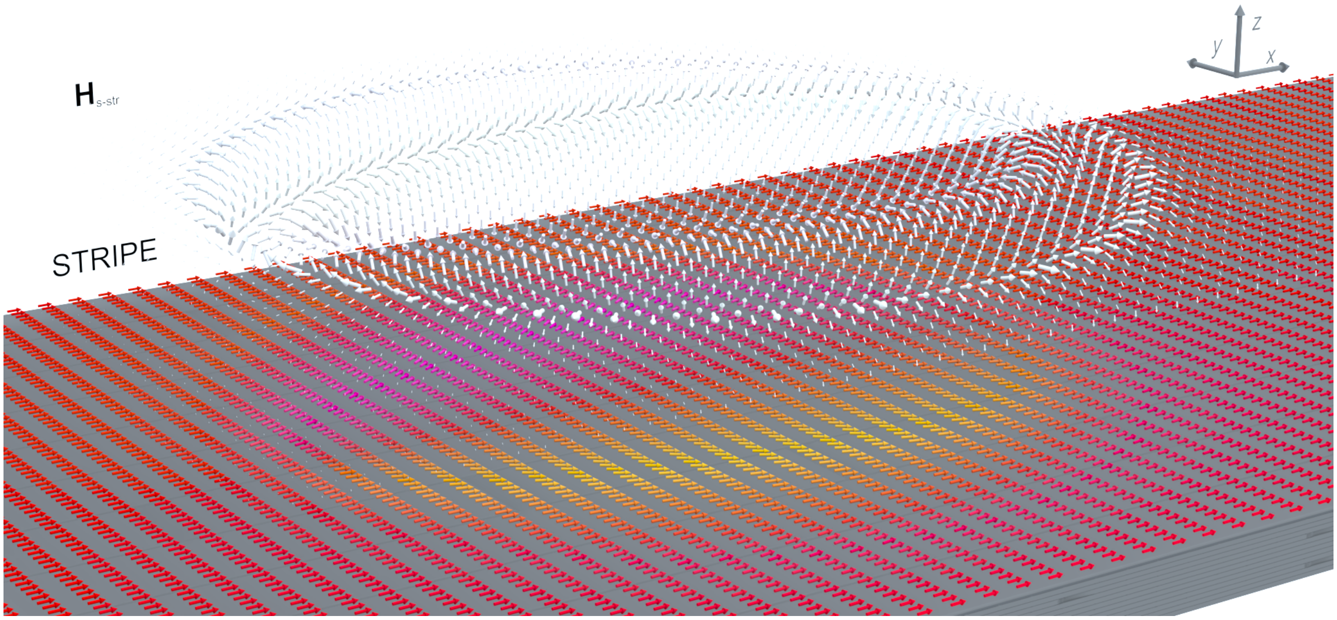

Figure S2: Magnetization in the Py stripe (colored arrows) and distribution of the stray magnetic field (gray arrows) generated solely by the imprint in the stripe, excluding the field from the nanodot. The size of the arrows is proportional to the magnetic field strength. The simulation performed with the freezing-spin technique with $D = -1.6$ mJ/m^2.

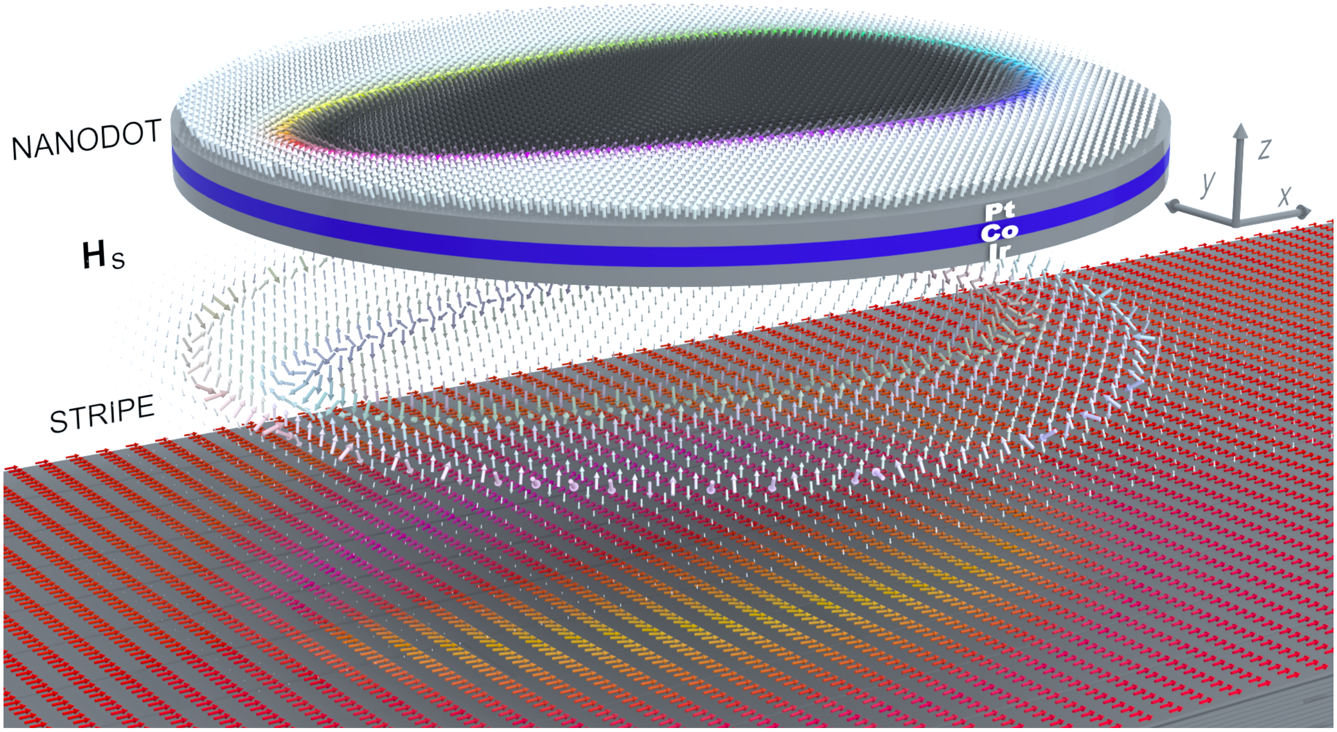

Figure S3: 3D plot of the magnetization texture and the stray magnetic field distribution in the investigated system for $D = -1.6$ mJ/m^2. The figure shows the spatial distribution of the magnetization in the nanodot (top), the magnetic field in the space between (middle), and the magnetization in the stripe (bottom). The color of the arrows in the stripe represents the direction of magnetization.

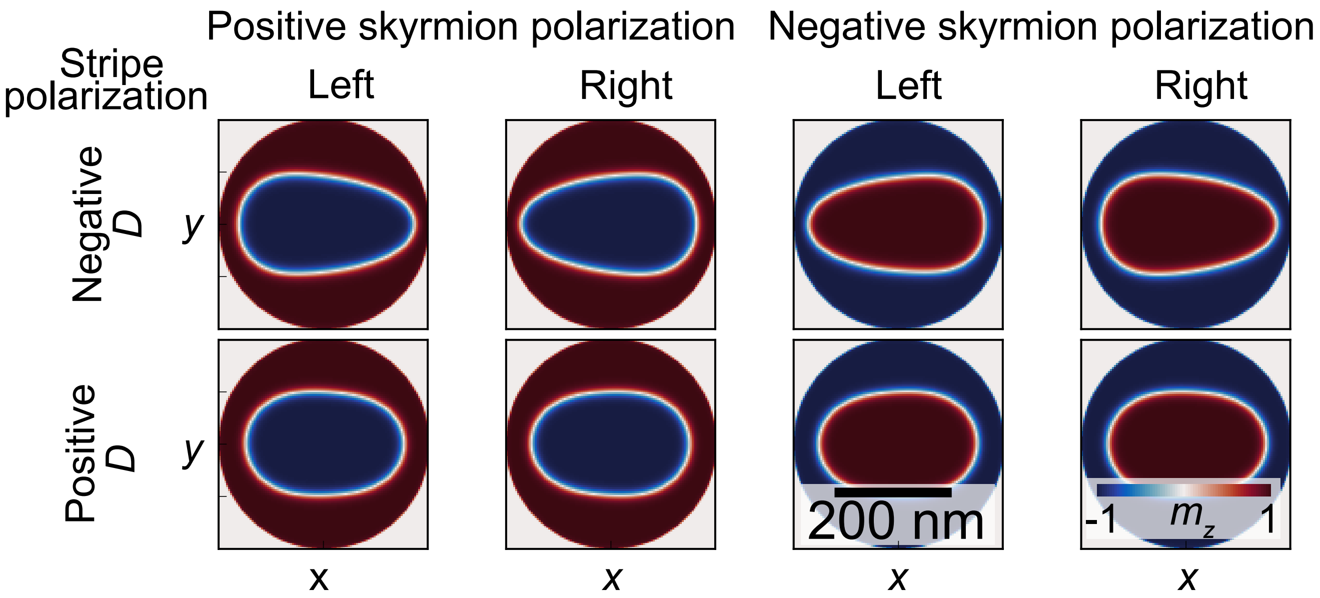

Figure S4: Static magnetization configuration in a Pt/Co/Ir nanodot with $D = \pm 1.6$ mJ/m^2 dipolarly coupled to the Py stripe. The color scale is given in reduced units of magnetization.

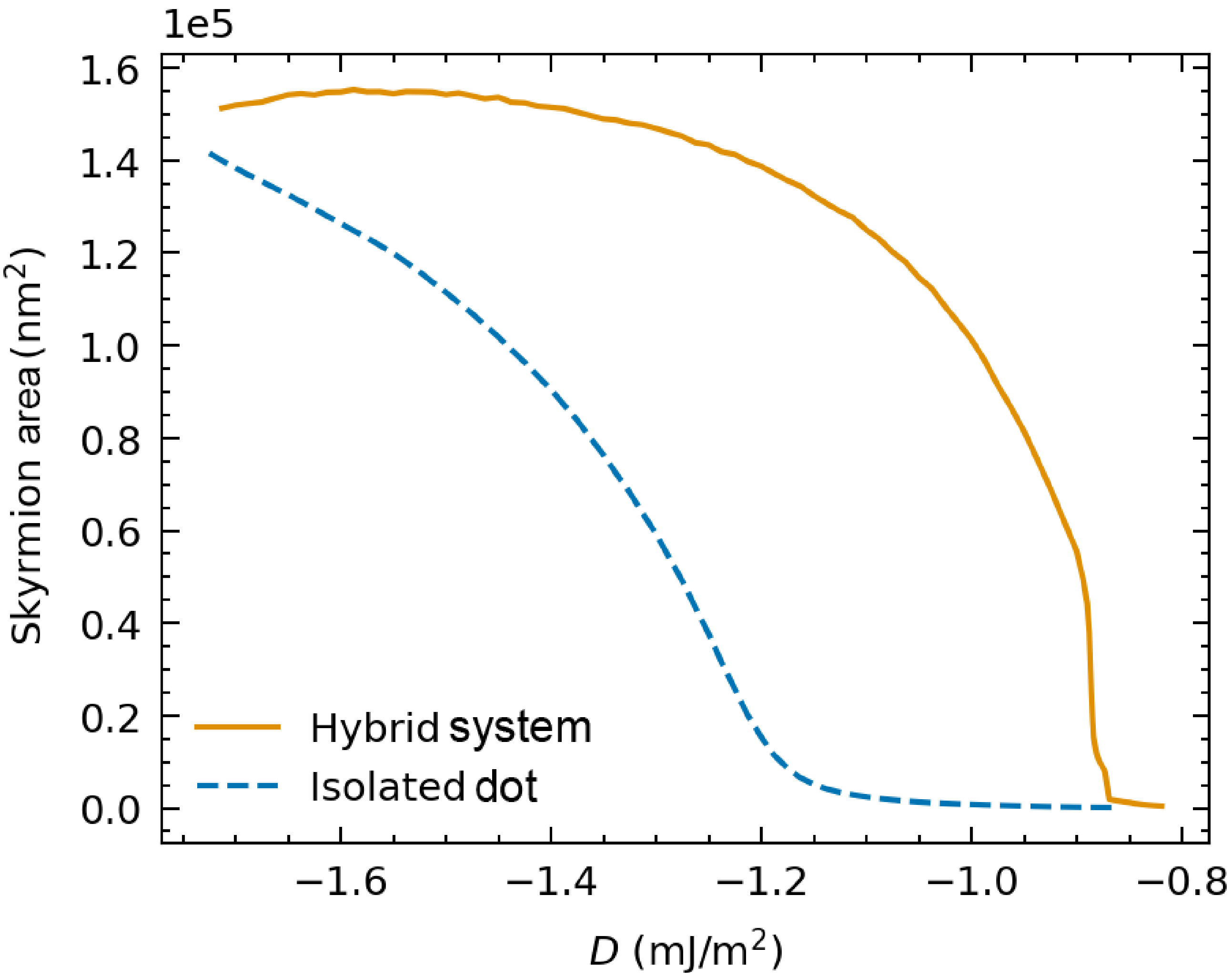

Figure S5: The dependence of the skyrmion area in the hybrid (solid orange line) and isolated system (dashed blue line) is shown in the figure. The area covered by the skyrmion across the entire range of negative DMI is larger than the skyrmion in an isolated system.

on the skyrmion's spatial characteristics.
